# Exploring one health-based strategies for rabies elimination: Overview and future prospects

**DOI:** 10.1371/journal.pntd.0013159

**Published:** 2025-06-18

**Authors:** Renyun Zha, Jianyun Lu, Jianying Chen, Cheng Guo, Jiahai Lu

**Affiliations:** 1 School of Public Health, Sun Yat-Sen University, Guangzhou, China; 2 Guangzhou Baiyun District Center for Disease Control and Prevention, Guangzhou, China; 3 One Health Center of Excellence for Research & Training, Sun Yat-Sen University, Guangzhou, China; 4 National Medical Products Administration Key Laboratory for Quality Monitoring and Evaluation of Vaccines and Biological Products, Guangzhou, China; 5 Hainan Key Novel Thinktank “Hainan Medical University ‘One Health’ Research Center”, Haikou, China; 6 Institute of One Health, Wenzhou Medical University, Wenzhou, China; 7 Key Laboratory of Tropical Diseases Control, Sun Yat-Sen University, Ministry of Education, Guangzhou, China; University of Abuja Faculty of Veterinary Medicine, NIGERIA

## Abstract

**Background:**

Establishing a comprehensive and coordinated mechanism for rabies management is essential for achieving the goal of eliminating the disease. It requires the involvement of multiple disciplines and departments, as well as the implementation of necessary policies and measures. The recent COVID-19 pandemic has added further challenges to the goal, particularly for developing countries like China. However, certain regions in China are leveraging local advantages and departmental strengths to actively explore effective strategies.

**Principal findings:**

This review provides an overview of the global prevalence of rabies, international cooperation efforts, and specific measures. Of particular significance is the analysis of the transformation of the rabies situation in China as well as an exemplar management of a rabies case in the Baiyun District of Guangzhou, Guangdong Province. We also discuss the hopeful action plan based on the One Health concept, aimed at achieving the goal of rabies elimination by 2030.

**Conclusions:**

Rabies remains a significant threat to public health and economies across most countries worldwide. Despite this, eliminating rabies is increasingly feasible, with China showcasing notable progress, including the adoption of the One Health approach in disease prevention and control strategies.

**Synopsis:**

The distinction between disease eradication and elimination lies in their scope and permanence. Eradication involves globally reducing the incidence of infection caused by a specific agent to zero, requiring no further intervention measures once achieved. In contrast, disease elimination focuses on reducing the incidence of infection within a specific geographic area to zero, necessitating ongoing actions to prevent its transmission or re-emergence. In the long history of humans’ battle against infectious diseases, the complete eradication of smallpox has undoubtedly been an inspiring achievement. However, emerging and re-emerging infectious diseases have remained in the forefront of people’s minds, causing significant morbidity, mortality, and potential economic burdens in impoverished countries and regions worldwide. It is disappointing that rabies has not been eradicated globally. While high-income countries have achieved the elimination of canine-mediated rabies through dog vaccination and population management programs, there are fewer examples of successful large-scale elimination of canine rabies in low- and middle-income countries, primarily limited to Latin America.

## Introduction

Rabies is an acute, lethal zoonotic infectious disease caused by rabies virus (RABV) infection, and is one of the oldest and most terrifying diseases known to humans [1]. The *Lyssavirus* rabies, a single negative-stranded RNA virus in the Rhabdoviridae family, invades the central nervous system through bites or scratches from infected animals or through contamination of fresh wounds or mucous membranes by infectious material [2,3]. Once a human is bitten by a rabid animal, the clinical manifestations are mainly manical. When clinical signs such as hydrophobia, photophobia, and laryngeal spasm emerge, the disease becomes usually fatal despite advanced medical interventions [4]. The *Lyssavirus* rabies can infect all mammals, however, the domestic dog is the principal reservoir host of the virus [5,6]. Over 7 million people worldwide are exposed to the *Lyssavirus* rabies due to dog bites every year, resulting in 59,000 deaths, and this number is considered to be an underestimate [7–9]. Unfortunately, most cases of rabies occur in the poorest and most vulnerable communities in the world [10]. Approximately 40% of the victims are children under the age of 15 living in Asia and Africa [11]. However, the 100% fatality of this disease is counterbalanced by its 100% preventability, which encompasses both individual and collective understanding. Research has shown that receiving rabies vaccination (with additional equine anti-rabies serum for severely bitten patients) following an animal bite can generate effective antibodies and prevent the occurrence of fatalities, regardless of the severity of the case [12]. A more realistic approach to prevention may be the goal of eliminating rabies, but this depends on the implementation of various necessary measures, including policies, economic considerations, and execution. For example, by implementing dog vaccination and population management programs, almost every high-income country has eliminated dog-mediated rabies [13]. Recent research on rabies has produced strong evidence to demonstrate the feasibility of eliminating canine rabies through large-scale vaccination of domestic dogs [14].

The International Task Force on the Elimination of Diseases (ITFDE) defines seven curable diseases and advises bodies such as the World Health Organization on various aspects of disease eradication [15]. Eradicating any infectious disease in the world is a daunting task, made even more challenging by the interplay of societal factors and the intrinsic characteristics of specific pathogens. The World Health Organization (WHO), the World Organization for Animal Health (OIE), the Food and Agricultural Organization of the United Nations (FAO), and the Global Alliance for Rabies Control (GARC) have established a global goal for the elimination of dog-mediated human rabies deaths by 2030 [16,17].

The COVID-19 pandemic, which erupted in 2019, has caused significant devastation to human society and will continue to have enduring impacts in the coming years. In fact, factors contributing to this outcome include the virus’s high transmissibility and variability, as well as policymakers’ strategies for post-COVID era response [18]. By comparison, despite varying measures and implementations for prevention and control during the COVID-19 pandemic across the globe, services for neglected tropical diseases (NTDs) were the most severely disrupted and the second most frequently disrupted by the pandemic [19]. Health services in low- and middle-income countries (LMICs), particularly in Africa and Asia, face significant challenges with exacerbated limitations on post-exposure prophylaxis (PEP). The control of rabies in Uganda has deteriorated due to funding deficits and the cancelation of mass dog vaccination, while other countries facing outbreaks have encountered difficulties in vaccine distribution, production, and research due to disrupted drug distribution and travel [20]. On the other side, however, the once-in-a-century COVID-19 pandemic appears to have some silver linings, as it has refocused attention on the threats at the interface of human-animal-environment, and it is expected to increase investments in “One Health” cooperation, which is a concentrated embodiment of cohesion, sustainable approaches, and evidence-based interventions in eliminating rabies [21]. Furthermore, we have gained the following insights: As demonstrated by the COVID-19 pandemic, effective prevention and response capacity building require the involvement of multiple stakeholders from different sectors and encourage their participation in decision-making. In addition, ensuring the effective implementation of policies and measures, rather than false or substandard practices, will be crucial for the success of rabies elimination programs.

### Global situation of rabies endemicity

Scattered monitoring data indicate that over 20,000 Africans die from rabies each year, with most cases occurring in rural areas with limited access to healthcare and limited monitoring [22,23]. Compared to the southern region, the western, eastern, and central regions are high-risk areas for rabies due to the lack or high cost of PEP and inadequate risk assessment [24–27]. Some countries have taken measures to control the situation, such as Kenya’s 15-year strategy to end dog-mediated human rabies deaths [28]. However, in many areas of Africa, further monitoring, prevention, and control measures are still needed to reduce the spread and death toll of the disease. Asian countries have taken a series of measures to prevent and control rabies, such as promoting dose- and cost-sparing intradermal (ID) vaccination, introducing identification card vaccination, and using anti-RABV monoclonal antibody vaccines [29,30]. Some countries have made significant progress, with South Korea declaring the elimination of local transmission of canine rabies and a significant reduction in rabies-related deaths in Sri Lanka, Bhutan, Thailand, and Bangladesh [31,32]. However, the entire Asian region still faces many challenges, including competing health priorities, a lack of inter-sector coordination and comprehensive rabies control programs, inadequate surveillance, and limited access to PEP [33]. The rabies situation in the Americas exhibits regional disparities. Despite overall progress in rabies control, challenges persist throughout the continent. In North America, encompassing Canada and the United States, significant advancements have been made through extensive dog vaccination campaigns, resulting in minimal reported human cases [34]. These countries have also implemented robust wildlife surveillance programs, effectively curbing the transmission of rabies from wildlife to humans. Mexico, Central America, and the Caribbean region have likewise achieved commendable progress in their efforts to control rabies. Mexico stands as an exemplary case, undertaking large-scale annual canine vaccination initiatives and earning recognition from the World Health Organization as a canine-mediated rabies-free nation [35,36]. Within the Caribbean region, only isolated pockets report instances of rabies, primarily transmitted through bats or mongooses [37]. Nevertheless, Haiti bears the greatest burden of rabies in this area and has implemented multifaceted measures to prevent and control the disease. Unfortunately, they have encountered setbacks due to environmental catastrophes, political instability, and the ongoing COVID-19 pandemic [38]. In South America, many countries have successfully achieved their targets of eliminating human rabies cases caused by dogs. Nevertheless, pockets of rabies transmission and outbreaks persist in certain regions, with Bolivia being particularly affected. Efforts to combat rabies in the Americas are ongoing, with various nations implementing comprehensive strategies aimed at further reducing the incidence of this deadly disease [39]. Continuous surveillance, collaboration, and effective implementation of preventive measures will be crucial in achieving sustained control over rabies throughout the continent. Undeniably, for a considerable period in the future, rabies will continue to pose significant risks to agriculture, public health, and conservation biology in both tropical and non-tropical regions (Table 1).

**Table 1:** Current situation and management experience of rabies around the globe.

Region	Situation	Iconic events	Limiting/promoting factors	Reference
Europe	Low incidence; Risk of travelers importing cases	Oral rabies vaccination programs have been adopted by all European countries since the late 1980s and are still ongoing.	Frequent tourism and communication bring input pressure; Regional conflicts may pose a risk of outbreak; The strong economic foundation of developed countries supports animal immunity.	[40]
Asia	Severe mortality and economic burden; Positive measures and actions are being implemented	A few developed countries (Japan, Singapore, South Korea) have adopted large-scale dog vaccination and stray dog population control plans; In 2004, the Asian Rabies Expert Bureau (AREB) was established.	Limited resource investment, including vaccine accessibility and improved monitoring systems; Lack of political commitment and departmental coordination; The public has a low level of education and weak awareness of prevention and control.	[41]
Africa	A large number of rabies deaths; Lack of accurate data	Tunisia has begun mass vaccination campaigns for dogs in the 1980s; In 2008, the Africa Rabies Expert Bureau (AfroREB) was established;	Insufficient understanding of rabies monitoring and burden; Lower national priority levels; Lack of sustainable plans for canine rabies control.	[42,43]
North America	A significant reduction in rabies cases in the United States and Mexico while Haiti still faces challenges	“Plan of Action for the Elimination of Urban Rabies from the Principal Cities of Latin America” (OPS, 1983)	The Pan American Health Organization (PAHO) coordinates canine vaccination, contact person treatment, and epidemiological monitoring.	[44]
South America	Most cases of rabies occur in countries such as Bolivia and Brazil			[45]
Oceania	Free of dog-mediated rabies but high risk of traveler’s cases	Australian Veterinary Emergency Plan (AUSVETPLAN) (Animal Health Australia, 2011)	Strict dog management and legal governance tools	[46,47]
Antarctica	Free of all lyssaviruses but with no laboratory-based surveillance	–	Uninhabited	[48]

Dog-mediated Rabies is an NTDs that poses a threat to practically every nation and area on Earth except for Oceania and Antarctica, where the disease is absent. Nonetheless, the geographic setting, the support of sources, the direction of policy, and public consciousness all impact the eradication of rabies.

### Policy priority and international cooperation

Eliminating rabies is a challenging yet highly valuable endeavor, as it not only benefits the health and well-being of residents but also serves as a national public good in the context of today’s globalized world. Indeed, achieving this goal requires a significant investment of public funds and leadership in developing an overall strategy for the elimination or control of rabies. It is also important to seek support from international organizations, public entities, private partners, and other stakeholders to bridge funding gaps and promote the development of sustainable rabies planning [49]. Absolutely, considering the limited financial resources and manpower of public health sectors in many countries, especially developing ones, it is crucial to prioritize the prevention and control of zoonotic diseases [50,51]. This necessitates the effective allocation of scarce funds and logistical resources towards activities aimed at reducing the outbreaks of rabies and minimizing the impact. At the national level, there is a need to reassess and adjust the prioritization of measures to ensure optimal practical outcomes. These activities encompass the vaccination and management of dogs, post-exposure prevention and treatment for humans, public awareness campaigns, animal surveillance, and various innovations in rabies technology. Under the ambitious goal of Zero by 2030, the Tripartite has seized the opportunity to launch the United Against Rabies Forum (UAR Forum) in 2020, aiming to establish a broad and inclusive network of stakeholders to collectively achieve the goal of rabies elimination [52]. One important initiative of this forum is to bring together organizations and departments from different countries, engaging in discussions and practical actions based on their respective needs and available resources [53]. For instance, the UAR forum is dedicated to advocating for the recognition of national strategic plans for rabies control by the World Organization for Animal Health (WOAH), and linking these plans with the agenda of the Global Alliance for Vaccines and Immunization (GAVI) regarding the accessibility of PEP [54,55].

### Vaccination and management of dogs

The lofty goal of achieving “Zero rabies deaths by 2030” can be accomplished through key actions such as large-scale dog vaccination campaigns and canine population management to eliminate rabies. Dogs act as maintenance hosts and reservoirs for canine rabies, and it is ideal to interrupt the transmission of the virus from dogs to other animals, including humans, as a preventive measure [56]. Large-scale dog vaccination campaigns are one of the most crucial actions for controlling rabies. In most Latin American countries, the health authorities coordinate these campaigns annually or biannually, with extensive intersectoral participation involving the veterinary services, communities and education sectors as part of the planning process [57]. Since 1983, the National Canine Vaccination Program, coordinated by the Pan American Health Organization (PAHO), has controlled canine rabies in most regions of the Western Hemisphere, reducing the incidence by over 99%. By focusing on large-scale vaccination of dogs, the control and elimination of this disease can be achieved with a vaccination coverage rate of 70%, a threshold that has been demonstrated through various studies [58–60]. Further support for this wise strategy lies in the undeniable economic cost-effectiveness of vaccinating dogs to prevent human deaths from rabies. A 6-year period cost-benefit analysis study conducted in Bhutan on mass dog vaccination versus human post-exposure treatment (PET) costs revealed that without a comprehensive dog vaccination program, relying solely on intensified human PET would impose significant direct healthcare expenses on the government [61]. Furthermore, even accounting for the increased time and resources associated with challenges in capturing stray dogs for vaccination, it is still much more cost-effective compared to the economic costs of performing sterilization surgeries on dogs [62,63]. Although culling dogs can more directly control the spread of the RABV, its effectiveness is limited [64,65]. Moreover, culling not only raises ethical concerns but may also compromise rabies prevention efforts. When animals are culled, their removal creates ecological vacancies that are rapidly filled by new animals with no immunization history. This replacement process perpetuates viral transmission by reintroducing susceptible individuals into the population [66]. However, achieving high coverage rates in dog vaccination campaigns remains a challenge for many countries around the world, leading to hesitancy and difficulties in implementation. Firstly, the success of such campaigns is closely tied to higher levels of logistical, political, and economic development. In many countries with endemic rabies, the lack of adequate logistical infrastructure hinders the implementation of vaccination programs that can achieve sufficient herd immunity (estimated at 70%) [17]. Furthermore, many veterinarians in Africa and Asia lack experience or confidence in handling dogs or implementing veterinary interventions involving pet dogs. This often results in inappropriate or ineffective vaccination or management of both owned and stray dogs [43]. Lastly, the effectiveness of mass dog vaccination campaigns requires a more comprehensive monitoring system involving multiple sectors, including human health and veterinary departments, to assess the actual impact achieved [57]. This monitoring helps to evaluate the effectiveness of the vaccination efforts and make necessary adjustments to ensure optimal outcomes. During the COVID-19 pandemic, rabies surveillance in countries where it is endemic has been weakened. In 25% of these countries, staff who conduct rabies surveillance have been reassigned to COVID-19 response efforts [20]. Combining efforts to improve immunization coverage and strengthen the management of dogs is essential, as it is evident that administering vaccines to dogs alone cannot achieve optimal results [67,68]. One crucial factor lies in the disparity of vaccine coverage between urban and rural areas, which consequently leads to a deficiency in overall immunization efficacy. Hence, it is imperative to implement a comprehensive set of measures aimed at mitigating the stray dog population. These measures encompass establishing dedicated shelters and organizing adoption initiatives, implementing comprehensive sterilization programs for stray dogs, regulating responsible ownership practices, and fostering public awareness campaigns [69]. Finally, oral rabies vaccination (ORV) may be the most cost-effective yet overlooked tool in the fight against rabies. Compared to conventional capture-vaccinate-release, ORV requires fewer human resources and expertise, particularly with regard to vaccinating wild animals where immunization can be challenging [70]. Attenuated live RABV vaccines and vector-based vaccines are currently the main types of ORV and have been proven to induce an immune response in dogs [71]. However, modified live virus vaccines and vector-based vaccines pose respective risks due to production process limitations: the former carries the potential for virulence reversion leading to rabies, while the latter may result in vector-related hazards and reduced efficacy [72].

### Post-exposure prophylaxis (PEP) and treatment

Although rabies is almost 100% fatal once symptoms appear, timely PEP can be effective in preventing both death and complications [7]. The earliest vaccine against rabies for humans was developed by Louis Pasteur in 1885, who tracked rabid rabbits and used their spinal cords as raw material, and such types of neurotropic vaccines were widely applied for a considerable period of time. Technological innovations have resulted in fewer adverse reactions and increased effectiveness of rabies vaccines. Since the 1950s, purified duck embryo vaccines (PDEVs), human diploid cell vaccine (HDCV), purified chick embryo cell vaccine (PCECV), primary hamster kidney cell vaccine (PHKCV), and purified Vero cell rabies vaccine (PVRV) have successively come into existence [73]. A meta-analysis of PCECVs currently administered via the IM or ID route for the PET showed that each dose of PCECV ≥ 2.5 IU can effectively prevent rabies [74]. Mentioned above and approved for use are all inactivated vaccines, and lower cost, shorter immunization procedures for attenuated live vaccines and alternative viral vector vaccines may be ideal alternatives in the future [75]. The emerging mRNA vaccines have recently attracted widespread attention, with a study showing that an optimized mRNA construct (LVRNA001) expressing the RABV-G protein provides dogs with a strong immune response and a 100% survival rate after live virus infection [76]. In cases of direct exposure, such as being bitten by a dog or contamination of skin or mucous membranes with animal saliva, the combined use of PEP and human rabies immune globulin (HRIG) is the optimal preventive measure [77]. The Rabies Post-Exposure Prophylaxis Guidelines from the Advisory Committee on Immunization Practices recommend that individuals with normal immune function and no previous vaccination receive PEP consisting of four doses of rabies vaccine and one dose of immune globulin, administered over a 2-week period [78]. Research has also indicated that in areas where rabies is prevalent and residents or travelers may have difficulty accessing timely and sufficient PEP, a single-visit Pre-Exposure Prophylaxis program appears to provide adequate protection for most healthy individuals without compromised immune function [79]. This approach would be beneficial in mitigating vaccine shortages and addressing the limited accessibility of high-cost HRIG. Access to human rabies PEP may be influenced by socioeconomic status, thereby posing challenges for achieving comprehensive coverage in many developing countries. In China, despite the availability of safer and more effective rabies vaccines, barriers such as high acquisition costs, limited financial subsidies, and inadequate community awareness hinder access to treatment for individuals at high risk of rabies [80,81].

### Post-vaccination evaluation of rabies vaccines

The serum rabies virus-neutralizing antibody (RVNA) is the primary indicator used to assess the effectiveness of rabies vaccines [82]. Current recommended quantitative detection methods for rabies-neutralizing antibodies by WHO and WOAH include the Rapid Fluorescent Focus Inhibition Test (RFFIT) and Fluorescent Antibody Virus Neutralization test (FAVN) [83]. The threshold level of RVNA is 0.5 IU/ml, which can be considered to induce an effective immune response against the virus. The National Disease Control and Prevention Administration of China has issued the “Guidelines for the Prevention and Disposal of Rabies Exposure (2023 Edition)”, which recommends that quarantine, veterinary and technical personnel, animal management and breeding personnel in the agricultural sector, laboratory personnel engaged in RABV work, medical personnel and caregivers caring for rabies patients, field workers, and tourists traveling to rabies epidemic areas should undergo pre-exposure immunization. Following the initial exposure, it is recommended to administer the rabies vaccine as early as possible. The immunization protocol should adhere to the latest nationally approved rabies vaccine products, which include two vaccination programs: a 5-dose regimen (administered on days 0, 3, 7, 14, and 28) and a “2-1-1” regimen (two doses on day 0, with each dose administered in the left and right deltoid muscles, followed by doses on days 7 and 21). In principle, rabies prevention and treatment clinics should be equipped with at least two different types of rabies vaccines. Corresponding management measures should be implemented based on the level of rabies exposure (Three grades: The Grade I involves wound cleansing only, the Grade II includes vaccination, and the Grade III necessitates both vaccination and passive immunization agents.). A systematic review and meta-analysis indicated that the human rabies vaccine for the Chinese population after PEP has good immunogenicity, with the RVNA level increasing to over 0.5 IU/mL 7 days after the administration [84]. Nonetheless, prior research has demonstrated that immunized dogs and cats have inadequate antibody titers, which may raise their risk of *Lyssavirus* rabies transmission [85,86]. This may be related to multiple factors, including vaccine type and batch, vaccination time and dosage, animal species, etc. [87]. Therefore, it is necessary to monitor RVNA for vaccinated animals and assess the risks to decide whether to strengthen vaccination. The concept of advancing the checkpoint included in One Health may suggest the need for regular antibody detection by vaccinated wildlife practitioners and pets to prevent rabies spillover.

### Animal surveillance and border control

**Fig 1:**
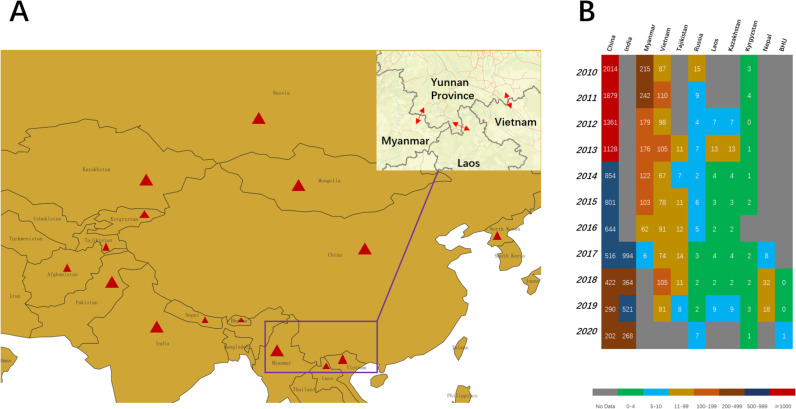
(A) Countries bordering China that are at high risk for rabies. (B) The number of rabies deaths in 11 countries including China, India, and Myanmar from 2010 to 2020. (The base layer of the map is from the U.S. Geological Survey, USGS, http://www.usgs.gov). (The risk categorization information is taken from the UK government website [98]; the triangle red mark indicates nations that are bordering China and are considered to be at high risk of contracting rabies.).

Excellent control strategies should be based on the establishment and improvement of a rabies surveillance and management system, complemented by the implementation of strict policy regulations, widespread public awareness campaigns, and education on prevention [88,89]. Two important groups, including domesticated animals and wild animals, are the primary hosts of the *Lyssavirus* rabies. Domesticated animals such as dogs and cats, and wild animals such as raccoons, skunks, bats, and foxes, respectively serve as vectors and reservoirs for the virus and form two interrelated cycles of transmission, namely urban and sylvatic cycles [90]. Indeed, simulations that bridge phylodynamics and spatial epidemiology suggest that human-mediated dispersal of infected dogs may continue to play a significant role in the spread of RABV within geographically established areas where the virus has been present for many years [91]. Additionally, international travel activities can also lead to potential rabies outbreaks as travelers may come into contact with rabid animals [92]. Therefore, conducting ongoing surveillance of animal rabies is crucial for controlling the disease at its source and disrupting transmission chains. Since 2004, China has initiated annual surveillance of animal rabies, supported by financial resources to facilitate animal diagnosis, monitoring, and vaccination activities [93]. Animal surveillance may play a crucial role in border control, as it is not only significant for the prevention and control of rabies in endemic countries but also has significant implications for the public health policies of regions where rabies has never occurred [94,95]. China is a multilateral country that borders up to 14 countries, all of which are high-risk countries for rabies. It is worth noting that Yunnan, a province in southern China that borders Myanmar, Laos, and Vietnam, experienced a large-scale outbreak of rabies in the 1980s (Fig 1A). Futhermore, the significant lack of data from countries such as India, Myanmar, Bhutan, and Nepal indicate highly inadequate rabies surveillance, posing potential risks of rabies transmission and outbreaks (Fig 1B). The aforementioned suggests that China should focus on emergency response plans, effective defensive barrier construction, and control of wildlife and dogs at national borders. Generally, geopolitical boundaries do not directly prevent the transmission of infectious diseases. However, it is common for countries to establish control measures focused on their borders. Zhenyang Guo et al. collected nucleoprotein sequences from samples isolated from various locations in Southeast Asia and analyzed their phylogenetic and geographical relationships, indicating that national geographical boundaries and border controls are effective barriers preventing the spread of rabies from China to adjacent regions [96]. Analysis of a series of transboundary raccoon-specific variants of the rabies virus (RRV) outbreaks in the eastern Canadian provinces that border the United States suggests that high local infection pressure may cause RRV to spread through the areas of vaccination across the international boundary, and this highlights the necessity of coordinated surveillance programs [97].

### Data-based publicity and education

A data-driven quantitative assessment is necessary for any disease control action that requires capital investment to consider affordability and the social-economic benefits it brings. Undoubtedly complex and requiring careful planning, the process of achieving the goal of eliminating rabies by 2030 can face a barrier to progress in the early stages due to a lack of data on the burden of rabies [99]. Therefore, organizing international experts to assess the national burden of rabies, developing a comprehensive monitoring system, and establishing a real-time cost-effectiveness model for control measures will provide clear and definitive guidance for the elimination of rabies [22,100,101]. In the 1980s, China introduced an important policy aimed at raising public awareness of rabies prevention and the behavior of rabid dogs to help high-risk populations, including economically disadvantaged rural areas, significantly reduce the risks associated with rabies [88]. In recent years, there has been a greater emphasis on the development of communication tools in the international community. Established through collaboration between the World Health Organization, the Food and Agriculture Organization, the World Organization for Animal Health, and the Global Alliance for Rabies Control, the “United Against Rabies” partnership aims to advocate for rabies control efforts globally [102]. The Pasteur Institute has developed courses for rabies control officials, training a significant number of advocates and experts, thereby strengthening countries’ capacity to develop and implement effective rabies control programs [103]. In addition, regional rabies awareness campaigns are widely being established. This includes the Pan-African Rabies Control Network (PARACON) and the ASEAN Rabies Elimination Strategy established in 2015. These initiatives aim to promote collaboration, information sharing, and coordinated efforts among countries within a specific region to eliminate rabies [49,100,104].

### Other innovations that are beneficial to the goal

In fact, the low laboratory diagnostic capacity for rabies in humans and animals has been a contributing factor to underreporting of cases and suboptimal surveillance outcomes. Although China has reported a large number of human and animal rabies cases, the majority have not been laboratory-confirmed and have only been diagnosed based on clinical presentations and retrospective epidemiologic surveys, which could potentially lead to confusion with other types of encephalitis or the neglect of asymptomatic carriers of the virus [105]. The direct fluorescent antibody test (DFAT), which has been certified by the WHO and OIE, has long been considered the global standard procedure for diagnosing rabies [106]. Other methods include enzyme-linked immunosorbent assay (ELISA), direct rapid immunohistochemical test (dRIT), and real-time reverse transcription-polymerase chain reaction (RT-PCR) [107]. Recently, a chip-based RT-PCR detection method called Truenat Rabies (Molbio Diagnostics) has emerged, which allows for semi-quantitative detection of the *Lyssavirus* rabies [108]. Truenat not only improves the sensitivity, specificity, and diagnostic accuracy of rabies detection but also is more accessible in most developing countries. With the rapid development of internet technology and a significant increase in the number of users, the surveillance of infectious diseases, especially zoonosis, is entering a new era. Due to the real-time and comprehensive nature of the internet, the collection of informal reports of human and animal diseases as well as environmental change data can be utilized to complement traditional monitoring tasks [109]. Internet biosurveillance, an internet-based tool developed and applied to achieve early warning and situational awareness of public health threats, has proven beneficial in triggering public health responses to mitigate infectious disease outbreaks [110]. A further example of practical application is during a rabies outbreak in the Kilombero Valley in 2007, where Katie Hampson and colleagues conceived a large-scale rabies surveillance system that was subsequently developed and deployed in southern Tanzania [111]. Since 2011, the mobile-phone-based surveillance system has been applied among frontline health and veterinary workers to collect a large amount of data on rabies exposure and requests for PEP, which holds significant value in improving the healthcare systems in LMICs.

### Rabies in China

China was previously considered a high-burden country for rabies, and the establishment of the National Infectious Disease Report Information System (NIDRIS) in 1950 facilitated the concrete reporting of rabies cases [112]. This system’s implementation provided a crucial foundation for accurate surveillance and monitoring of rabies cases, thus promoting the advancement of rabies control efforts in China. Rabies, as a legally class II notifiable infectious disease in China, recorded nearly 108,412 cases from 1950 to 2004, exhibiting three distinct peaks, with the highest reaching 7,037 cases [113]. From 2005 to 2012, 30 provinces in China reported 19,221 cases of human and animal rabies, primarily concentrated in rural areas of the southern and eastern regions [114]. Subsequently, there has been an unprecedented reduction in the number of rabies cases on the Chinese mainland, with only 136 cases reported in 2021, and this number may even decrease to double digits in 2022. Undoubtedly, this reduction could be attributed to the strict control measures implemented during the COVID-19 pandemic, which have minimized opportunities for human contact with infected animals. Subsequently, several significant outbreaks of rabies occurred in mainland China, but fortunately, the Chinese government proposed effective policies that gradually reduced the number of cases [115]. China has unique national conditions, with a massive population base and vast areas of population residence, which have resulted in the significant economic burden of vaccination. Furthermore, the urbanization process has also exacerbated difficulties in managing and monitoring dogs [116]. It is worth noting that the decreased number of rabies cases in mainland China can be attributed to the increased availability of efficacious and safe cell-culture rabies vaccines rather than the implementation of dog control measures [117]. By comparison, a recent study that utilized the updated national data to predict the future burden of human rabies and evaluate the cost-effectiveness of comprehensive rabies control strategies in China suggests that expanding the vaccination coverage of dogs, rather than increasing the accessibility of PEP, is crucial to achieving China’s elimination goal as early as 2033 [118]. Indeed, this change in approach reflects a shift in the situation and strategy of rabies prevention and control in China. As a result, the Chinese government and relevant organizations have taken a series of control strategies and measures to actively respond to the current rabies situation in China. Currently, China adopts a joint mechanism to combat rabies, with the government taking the lead, multiple departments coordinating efforts, and active participation from the entire society. In line with international guidelines, China has implemented key measures for rabies control, including canine management, PEP and treatment for humans, animal monitoring and reporting, as well as extensive societal-level awareness campaigns [119]. As the largest developing country, China faces the challenge of vaccinating nearly 60 million dogs in rural areas, which puts a strain on its immunization planning capacity. Additionally, the issue of substandard quality rabies vaccines for animals, along with the low income levels prevalent in rural areas, greatly hinders the expansion of vaccine coverage rates [120].It is not surprising to observe that the prevalence of *Lyssavirus* rabies neutralizing antibody (VNA) positivity is significantly higher in developed regions of China compared to rural areas, and this discrepancy reflects the imbalance in the risk of rabies outbreaks and the focus of control efforts [121]. In addition, inadequate awareness education makes it difficult to ensure that patients receive appropriate or timely wound treatment, PEP, or rabies immunoglobulin after exposure [122].

### Baiyun strategy: A case of rabies disposal

Guangdong Province may be one of the provinces in China with the most significant changes in the rabies situation, as it was once a high-incidence province and had an impact on neighboring provinces and even the national epidemic. From 2007 to 2011, Guangdong Province ranked second in the country in terms of the number of rabies cases, reaching 1,485, second only to Guangxi with 1,778 cases [123]. In stark contrast, from 2016 to 2020, Guangdong Province reported only 99 cases of rabies. Guangzhou, a crucial and diverse city in southern China and also the capital of Guangdong Province, encompasses both rural and urban administrative areas. In the process of rabies prevention and control, Guangzhou has undergone challenging but relatively successful exploration, leading to the establishment of a collaborative and comprehensive rabies control system involving multiple departments [88]. Proudly supporting this assertion are the remarkable data from 2014 and 2015, during which there were no reported cases in Guangzhou. This experience provides valuable lessons for other countries and regions facing similar rabies challenges. Baiyun district in the northern part of Guangzhou’s old town has a registered population of 1.2 million people, but a significantly higher resident population of 3.6 million people, mainly migrant workers, posing risks for zoonotic disease transmission like rabies. However, as a key area of focus for rabies prevention and control in Guangzhou, the Baiyun District Center for Disease Prevention and Control has developed its own unique management system. The following is a detailed account of the handling process under the “Baiyun Model” based on the preliminary epidemiological investigation report regarding a suspected case of rabies in Jiahe Street, Baiyun District.

On April 5th, 2023, a suspected case of rabies was reported to Baiyun CDC by Guangzhou Medical University Affiliated Hospital (Hospital Eight), where the patient was admitted. The center immediately launched an emergency response and began investigating the case. The investigation revealed that the patient had been keeping four cats in a factory, one of which had gone missing, and then returned on April 1st with symptoms of illness, scratching and biting the patient. The patient subsequently went to Hospital Eight for the rabies vaccination and the immunoglobulin injection before returning home. On April 4th, the patient developed symptoms such as fear of wind and water, difficulty breathing, fever with the highest temperature at 37.5 °C, muscle pain, mental tension, poor appetite, and sleep. On April 6th, the provincial CDC laboratory reported that the nucleic acid test results of the patient’s sputum and cerebrospinal fluid samples for *Lyssavirus* rabies were negative, while another sample tested positive using second-generation gene sequencing at Hospital Eight’s laboratory. On April 7th, Hospital Eight collected new samples of the patient’s saliva and cerebrospinal fluid and sent them to the Guangdong Provincial CDC for *Lyssavirus* rabies nucleic acid testing. The saliva sample tested strongly positive for RABV nucleic acid, while the cerebrospinal fluid sample tested weakly positive.

Upon receiving the case report, the center promptly reported it to the higher-level administrative and professional departments. An immediate case investigation and a suspected exposure investigation were initiated, and terminal disinfection was carried out at the patient’s residence and workplace. Saliva and cerebrospinal fluid specimens were collected from the case and temporarily stored at the laboratory of the Eighth Hospital since the provincial CDC was unable to receive the samples on April 5. The patient’s five family members and employees have been vaccinated against rabies. In addition to receiving the rabies vaccine, the wife and employees have also received rabies immunoglobulin, while the others have not. Ten medical staff involved in the treatment at Jiahe Yimin Hospital were equipped with personal protective measures throughout the treatment process and were vaccinated against rabies on April 5 (without receiving rabies immunoglobulin). It is recommended that the 13 medical staff involved in the treatment at the Eighth Hospital be vaccinated with both the rabies vaccine and immunoglobulin. The local street authority investigated the factory and surrounding residents of the case and recommended that residents who were bitten by cats or dogs 10 days ago should receive rabies vaccination. The Health Department of Baiyun District investigated and found that in the past week, a total of 11 patients with Class III exposure who were bitten by stray dogs were treated at three animal bite treatment clinics located near the epidemic site. These patients have been notified to receive rabies immunoglobulin. On April 5th, the Agriculture and Forestry Department of the district disposed of the remaining two animals involved in the outbreak and collected brain tissue samples from these animals. The samples were sent to relevant units of the Guangdong Provincial Academy of Agricultural Sciences for *Lyssavirus* rabies pathogen testing, and the results were negative. On April 6th, the Agricultural and Rural Bureau of Baiyun District administered veterinary rabies vaccines to 149 dogs, cats, and other animals in Changhong Village near the epidemic site. A comprehensive campaign to prevent rabies has been carried out throughout the district, including the dissemination of preventive knowledge and the promotion of responsible pet ownership. Residents are educated on the importance of reporting stray dogs and cats promptly. In the event of an accidental animal bite or scratch, individuals are advised to proactively seek treatment at nearby rabies disposal clinics for human rabies vaccination and immunoglobulin prophylaxis to reduce the risks. A total of 15,678 informational leaflets have been distributed, reaching 6,895 households through door-to-door campaigns and being forwarded to 10 resident groups online.

**Fig 2:**
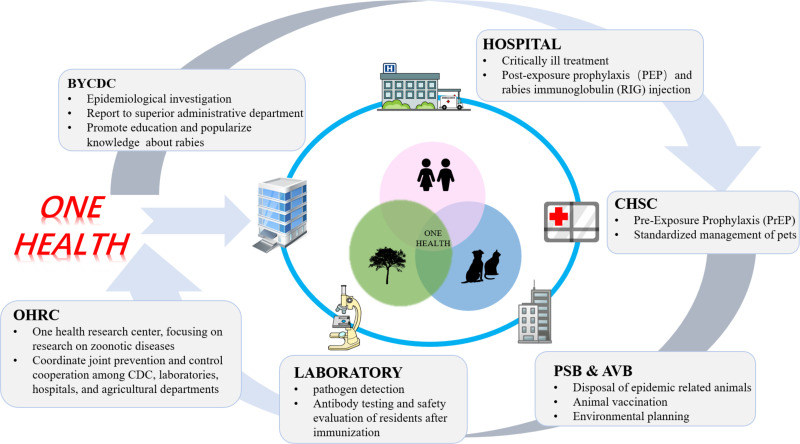
A multi-sectoral coordination mechanism for rabies prevention and control, established under the One Health framework. OHRC, One Health Research Center; BYCDC, Baiyun District Center for Disease Prevention and Control; CHSC, Community Health Service Centers; PSB&AVB, Public Security Bureau and Agricultural Veterinary Bureau. (Open Clipart (https://openclipart.org/) under Creative Commons Zero 1.0 Public Domain License.).

The handling process of this case witnessed a closely-knit and highly efficient joint prevention and control network formed by the CDC, hospitals, the Agricultural Veterinary Bureau, the Public Security Bureau, laboratories, Community Health Service Centers, and the local street authority. It is noteworthy that the One Health Research Center (OHRC) at Sun Yat-sen University provided professional guidance in the field of zoonotic disease prevention and control, while the Key Laboratory for Quality Monitoring and Evaluation of Vaccines and Biological Products (NMPA) and the Baiyun CDC signed a strategic cooperation agreement, offering relevant support. Under the guidance of OHRC at Sun Yat-sen University, BYCDC has developed a new model of joint prevention and control of rabies with multiple departments, such as the agricultural department, community health service centers, hospitals, and the laboratory (Fig 2). On a macro level, NMPA & OHRC is a professional academic research center for one health, coordinating cooperation among various departments and conducting interdisciplinary, cross-departmental, and cross-regional exchange activities. This interdisciplinary, interdepartmental, and interregional collaboration, which aligns with the One Health concept, has played a crucial role in reducing the cost of rabies control and elimination as well as enhancing efficiency [124–126]. However, unexpected issues arose during the handling process of this case. Even though canines are the primary hosts of rabies, especially in urban areas, it was surprising that the culprit in this case was a cat [127,128]. This, to some extent, reflects insufficient animal surveillance efforts and limited coverage of rabies vaccination among animals. Given the ongoing process of urbanization, the urban populations of both domestic and stray cats have witnessed a precipitous rise, consequently amplifying associated risks to public health. While the goal of “Elimination Rabies By 2030” prioritizes interventions targeting canine-mediated occurrences and transmissions, the potential contribution of other animal reservoirs, notably feral cats, is often underestimated. Through concerted collaborative efforts, it is imperative to acknowledge that the realization of this overarching goal hinges upon the comprehensive incorporation of multifaceted uncertainties. Therefore, both scientific research entities and public health agencies are urged to intensify their focus on expanding the scope of preventive measures and surveillance protocols targeting infectious reservoirs. Additionally, there was a significant delay in reporting the case from the time of the incident to the manifestation of symptoms, potentially resulting in serious consequences [3]. Therefore, it is necessary to establish a comprehensive system for vaccine administration registration and timely reporting, as well as organizing assessments and providing recommendations [129]. These measures would be ideal for completing the last mile in ensuring effective control and prevention of rabies.

## Conclusion

Rabies continues to pose a threat to the health and lives of people in the vast majority of countries worldwide, causing incalculable economic burdens. However, eliminating rabies is not an impossible task; on the contrary, it is a highly achievable goal as more and more countries are joining the effort. China, once an area severely affected by rabies as a developing country, has made significant improvements in recent years. Throughout the entire effort to combat rabies, numerous invaluable experiences are worthy of reference., including the One Health concept that guides strategies for zoonotic disease prevention and control, which is being implemented in many regions across China.

**Key learning points**

- **The One Health strategy is crucial for achieving the goal for the elimination of dog-mediated human rabies deaths by 2030.**
- **Large-scale vaccination and management of dogs serve as core interventions.**
- **Cross-border transmission risks necessitate enhanced coordinated surveillance.**
- **Animal surveillance should prioritize attention to the diversity of potential animal reservoirs.**

**Top five papers**

1. **WHO. Global framework to eliminate human rabies transmitted by dogs by 2030. Geneva: WHO; 2016.**
2. **Acharya KP, Subedi D, Wilson RT. Rabies control in South Asia requires a One Health approach. One Health. 2021;12:100215.**
3. **Octaria R, Salyer SJ, Blanton J, Pieracci EG, Munyua P, Millien M, et al. From recognition to action: A strategic approach to foster sustainable collaborations for rabies elimination. PLoS Negl Trop Dis. 2018;12(**10**):e0006756.**
4. **Yin W, Fu ZF, Gao GF. Progress and Prospects of Dog-Mediated Rabies Elimination in China. China CDC Wkly. 2021;3(**39**):831-4.**
5. **Tan J, Wang R, Ji S, Nanjing Agricultural University research group of The Challenge Cup Rabies Research G, Su S, Zhou J. One Health strategies for rabies control in rural areas of China. Lancet Infect Dis. 2017;17(**4**):365-7.**

## References

[1] Zhang J, Jin Z, Sun G-Q, Zhou T, Ruan S. Analysis of rabies in China: transmission dynamics and control. PLoS One. 2011;6(7):e20891. doi: 10.1371/journal.pone.0020891 21789166 PMC3137549

[2] Badrane H, Tordo N. Host switching in Lyssavirus history from the Chiroptera to the Carnivora orders. J Virol. 2001;75(17):8096–104. doi: 10.1128/jvi.75.17.8096-8104.2001 11483755 PMC115054

[3] Hemachudha T, Ugolini G, Wacharapluesadee S, Sungkarat W, Shuangshoti S, Laothamatas J. Human rabies: neuropathogenesis, diagnosis, and management. Lancet Neurol. 2013;12(5):498–513. doi: 10.1016/S1474-4422(13)70038-3 23602163

[4] Abdulmajid S, Hassan AS. Analysis of time delayed rabies model in human and dog populations with controls. Afr Mat. 2021;32(5–6):1067–85.

[5] Hueffer K, Murphy M. Rabies in Alaska, from the past to an uncertain future. Int J Circumpolar Health. 2018;77(1):1475185. doi: 10.1080/22423982.2018.1475185 29764319 PMC7011961

[6] LeRoux K, Stewart D, Perrett KD, Nel LH, Kessels JA, Abela-Ridder B. Rabies control in KwaZulu-Natal, South Africa. Bull World Health Organ. 2018;96(5):360–5. doi: 10.2471/BLT.17.194886 29875521 PMC5985419

[7] Smreczak M, Żmudziński JF. Current threat of rabies in Europe and in the world. Medycyna Weterynaryjna. 2019;75(01):6179–2019. doi: 10.21521/mw.6179

[8] Knobel DL, Cleaveland S, Coleman PG, Fèvre EM, Meltzer MI, Miranda MEG, et al. Re-evaluating the burden of rabies in Africa and Asia. B World Health Organ. 2005;83(5):360–8. PMC2626230 15976877

[9] Ehrenberg N, Ehrenberg JP, Fontes G, Gyapong M, Rocha EMM, Steinmann P. Neglected tropical diseases as a barometer for progress in health systems in times of COVID-19. BMJ Global Health. 2021;6(4). 10.1136/bmjgh-2020-004709 PMC8050874 33849898

[10] Chen Y, Tian J, Chen J-L. Challenges to eliminate rabies virus infection in China by 2020. Lancet Infect Dis. 2017;17(2):135–6. doi: 10.1016/S1473-3099(16)30589-8 28017558

[11] Cleaveland S, Fèvre EM, Kaare M, Coleman PG. Estimating human rabies mortality in the United Republic of Tanzania from dog bite injuries. Bull World Health Organ. 2002;80(4):304–10. 12075367 PMC2567765

[12] Warrell MJ, Nicholson KG, Warrell DA, Suntharasamai P, Chanthavanich P, Viravan C, et al. Economical multiple-site intradermal immunisation with human diploid-cell-strain vaccine is effective for post-exposure rabies prophylaxis. Lancet. 1985;1(8437):1059–62. doi: 10.1016/s0140-6736(85)92367-0 2860284

[13] Velasco-Villa A, Escobar LE, Sanchez A, Shi M, Streicker DG, Gallardo-Romero NF, et al. Successful strategies implemented towards the elimination of canine rabies in the Western Hemisphere. Antiviral Res. 2017;143:1–12. doi: 10.1016/j.antiviral.2017.03.023 28385500 PMC5543804

[14] Cleaveland S, Hampson K. Rabies elimination research: juxtaposing optimism, pragmatism and realism. P Roy Soc B-Biol Sci. 2017;284(1869). 10.1098/rspb.2017.1880 PMC5745407 29263285

[15] International Task Force for Disease Eradication; 2023. Available from: https://www.cartercenter.org/health/itfde/index.html.

[16] WHO. Global framework to eliminate human rabies transmitted by dogs by 2030; 2016. [cited 2023 Dec 6]. Available from: https://www.who.int/news/item/16-03-2016-global-framework-to-eliminate-human-rabies-transmitted-by-dogs-by-2030.

[17] Wallace RM, Undurraga EA, Blanton JD, Cleaton J, Franka R. Elimination of dog-mediated human rabies deaths by 2030: needs assessment and alternatives for progress based on dog vaccination. Front Vet Sci. 2017;4:9. 28239608 10.3389/fvets.2017.00009 PMC5300989

[18] Telenti A, Arvin A, Corey L, Corti D, Diamond MS, García-Sastre A, et al. After the pandemic: perspectives on the future trajectory of COVID-19. Nature. 2021;596(7873):495–504. doi: 10.1038/s41586-021-03792-w 34237771

[19] Goel K, Sen A, Satapathy P, Asumah MN, John OO, Padhi BK, et al. Rabies on rise in Africa amid COVID and monkeypox: a global health concern. QJM. 2023;116(7):594–6. doi: 10.1093/qjmed/hcac266 36448691

[20] Nadal D, Beeching S, Cleaveland S, Cronin K, Hampson K, Steenson R, et al. Rabies and the pandemic: lessons for One Health. Trans R Soc Trop Med Hyg. 2022;116(3):197–200. doi: 10.1093/trstmh/trab123 34392375 PMC8890778

[21] Mwatondo A, Rahman-Shepherd A, Hollmann L, Chiossi S, Maina J, Kurup KK, et al. A global analysis of One Health Networks and the proliferation of One Health collaborations. Lancet. 2023;401(10376):605–16. doi: 10.1016/S0140-6736(22)01596-3 36682370

[22] Hampson K, Coudeville L, Lembo T, Sambo M, Kieffer A, Attlan M, et al. Estimating the global burden of endemic canine rabies. PLoS Negl Trop Dis. 2015;9(4):e0003709. doi: 10.1371/journal.pntd.0003709 25881058 PMC4400070

[23] Mshelbwala PP, Weese JS, Sanni-Adeniyi OA, Chakma S, Okeme SS, Mamun AA, et al. Rabies epidemiology, prevention and control in Nigeria: scoping progress towards elimination. PLoS Negl Trop Dis. 2021;15(8):e0009617. doi: 10.1371/journal.pntd.0009617 34398902 PMC8389847

[24] Diallo MK, Diallo AO, Dicko A, Richard V, Espie E. Human rabies post exposure prophylaxis at the Pasteur Institute of Dakar, Senegal: trends and risk factors. BMC Infect Dis. 2019;19(1):321. 30975098 10.1186/s12879-019-3928-0 PMC6460513

[25] Changalucha J, Steenson R, Grieve E, Cleaveland S, Lembo T, Lushasi K, et al. The need to improve access to rabies post-exposure vaccines: lessons from Tanzania. Vaccine. 2019;37 Suppl 1(Suppl 1):A45–53. doi: 10.1016/j.vaccine.2018.08.086 30309746 PMC6863039

[26] Athingo R, Tenzin T, Shilongo A, Hikufe E, Shoombe KK, Khaiseb S, et al. Fighting dog-mediated rabies in Namibia-implementation of a rabies elimination program in the northern communal areas. Trop Med Infect Dis. 2020;5(1). 10.3390/tropicalmed5010012 PMC7157552 31963400

[27] Mbilo C, Kabongo J-B, Pyana PP, Nlonda L, Nzita RW, Luntadila B, et al. Dog ecology, bite incidence, and disease awareness: a cross-sectional survey among a rabies-affected community in the Democratic Republic of the Congo. Vaccines (Basel). 2019;7(3):98. doi: 10.3390/vaccines7030098 31454908 PMC6789516

[28] Gongal G, Wright AE. Human rabies in the WHO Southeast Asia Region: forward steps for elimination. Adv Prev Med. 2011;2011:383870. doi: 10.4061/2011/383870 21991437 PMC3178116

[29] Gongal G, Sampath G. Introduction of intradermal rabies vaccination—A paradigm shift in improving post-exposure prophylaxis in Asia. Vaccine. 2019;37(1):A94–8. 10.1016/j.vaccine.2018.08.034 30150166

[30] Gogtay NJ, Munshi R, Ashwath Narayana DH, Mahendra BJ, Kshirsagar V, Gunale B, et al. Comparison of a novel human rabies monoclonal antibody to human rabies immunoglobulin for postexposure prophylaxis: a phase 2/3, randomized, single-blind, noninferiority, controlled study. Clin Infect Dis. 2018;66(3):387–95. doi: 10.1093/cid/cix791 29020321

[31] Yang D-K, Kim H-H, Lee K-K, Yoo J-Y, Seomun H, Cho I-S. Mass vaccination has led to the elimination of rabies since 2014 in South Korea. Clin Exp Vaccine Res. 2017;6(2):111–9. doi: 10.7774/cevr.2017.6.2.111 28775975 PMC5540959

[32] Acharya KP, Subedi D, Wilson RT. Rabies control in South Asia requires a One Health approach. One Health. 2021;12:100215. doi: 10.1016/j.onehlt.2021.100215 33681445 PMC7907975

[33] Rupprecht CE, Mani RS, Mshelbwala PP, Recuenco SE, Ward MP. Rabies in the tropics. Curr Trop Med Rep. 2022;9(1):28–39. doi: 10.1007/s40475-022-00257-6 35371908 PMC8960221

[34] Ma X, Monroe BP, Wallace RM, Orciari LA, Gigante CM, Kirby JD, et al. Rabies surveillance in the United States during 2019. J Am Vet Med Assoc. 2021;258(11):1205–20. doi: 10.2460/javma.258.11.1205 33978439

[35] Del Rio Vilas VJ, Freire de Carvalho MJ, Vigilato MAN, Rocha F, Vokaty A, Pompei JA, et al. Tribulations of the last mile: sides from a regional program. Front Vet Sci. 2017;4:4. doi: 10.3389/fvets.2017.00004 28197407 PMC5281541

[36] Gonzalez-Roldan JF, Undurraga EA, Meltzer MI, Atkins C, Vargas-Pino F, Gutierrez-Cedillo V. Cost-effectiveness of the national dog rabies prevention and control program in Mexico, 1990-2015. PLoS Negl Trop Dis. 2021;15(3):e0009130. 10.1371/journal.pntd.0009130 PMC7963054 33661891

[37] Seetahal JFR, Vokaty A, Vigilato MAN, Carrington CVF, Pradel J, Louison B. Rabies in the caribbean: a situational analysis and historic review. Trop Med Infect Dis. 2018;3(3). 10.3390/tropicalmed3030089 PMC6160905 30274485

[38] Wallace R, Etheart M, Ludder F, Augustin P, Fenelon N, Franka R. The health impact of rabies in Haiti and recent developments on the path toward elimination, 2010-2015. Am J Trop Med Hyg. 2017;97(4):76–83. 10.4269/ajtmh.16-0647 PMC5676638 29064363

[39] Freire de Carvalho M, Vigilato MAN, Pompei JA, Rocha F, Vokaty A, Molina-Flores B. Rabies in the Americas: 1998-2014. PLoS Negl Trop Dis. 2018;12(3):e0006271. 10.1371/journal.pntd.0006271 PMC5877887 29558465

[40] Riccardi N, Giacomelli A, Antonello RM, Gobbi F, Angheben A. Rabies in Europe: an epidemiological and clinical update. Eur J Intern Med. 2021;88:15–20. doi: 10.1016/j.ejim.2021.04.010 33934971

[41] Tenzin, Ward MP. Review of rabies epidemiology and control in South, South East and East Asia: past, present and prospects for elimination. Zoonoses Public Health. 2012;59(7):451–67. doi: 10.1111/j.1863-2378.2012.01489.x 23180493

[42] Dodet B, Africa Rabies Bureau (AfroREB). The fight against rabies in Africa: from recognition to action. Vaccine. 2009;27(37):5027–32. doi: 10.1016/j.vaccine.2009.06.030 19560430

[43] Lembo T, Hampson K, Kaare MT, Ernest E, Knobel D, Kazwala RR, et al. The feasibility of canine rabies elimination in Africa: dispelling doubts with data. PLoS Negl Trop Dis. 2010;4(2):e626. doi: 10.1371/journal.pntd.0000626 20186330 PMC2826407

[44] Belotto A, Leanes LF, Schneider MC, Tamayo H, Correa E. Overview of rabies in the Americas. Virus Res. 2005;111(1):5–12. 15896398 10.1016/j.virusres.2005.03.006

[45] Ruiz M, Chávez CB. Rabies in Latin America. Neurol Res. 2010;32(3):272–7. doi: 10.1179/016164110X12645013284257 20406605

[46] Mills DJ, Lau CL, Weinstein P. Animal bites and rabies exposure in Australian travellers. Med J Aust. 2011;195(11–12):673–5. 22171863 10.5694/mja10.11413

[47] Degeling C, Brookes V, Lea T, Ward M. Rabies response, One Health and more-than-human considerations in Indigenous communities in northern Australia. Soc Sci Med. 2018;212:60–7. doi: 10.1016/j.socscimed.2018.07.006 30005225

[48] Rupprecht CE, Bannazadeh Baghi H, Del Rio Vilas VJ, Gibson AD, Lohr F, Meslin FX, et al. Historical, current and expected future occurrence of rabies in enzootic regions. Rev Sci Tech. 2018;37(2):729–39. doi: 10.20506/rst.37.2.2836 30747113

[49] Octaria R, Salyer SJ, Blanton J, Pieracci EG, Munyua P, Millien M, et al. From recognition to action: a strategic approach to foster sustainable collaborations for rabies elimination. PLoS Negl Trop Dis. 2018;12(10):e0006756. doi: 10.1371/journal.pntd.0006756 30359378 PMC6201874

[50] Rist CL, Arriola CS, Rubin C. Prioritizing zoonoses: a proposed one health tool for collaborative decision-making. PLoS One. 2014;9(10):e109986. doi: 10.1371/journal.pone.0109986 25302612 PMC4193859

[51] Pieracci EG, Hall AJ, Gharpure R, Haile A, Walelign E, Deressa A. Prioritizing zoonotic diseases in Ethiopia using a one health approach. One Health. 2016;2:131–5. 28220151 10.1016/j.onehlt.2016.09.001 PMC5315415

[52] Tidman R, Thumbi SM, Wallace R, de Balogh K, Iwar V, Dieuzy-Labaye I, et al. United against rabies forum: the One Health concept at work. Front Public Health. 2022;10:854419. 35493394 10.3389/fpubh.2022.854419 PMC9043483

[53] Tidman R, Fahrion AS, Thumbi SM, Wallace RM, De Balogh K, Iwar V, et al. United against rabies forum: the first 2 years. Front Public Health. 2023;11:1010071. doi: 10.3389/fpubh.2023.1010071 37033019 PMC10076768

[54] WHO, FAO, WOAH. United against rabies forum 2021 review 2021 [updated 27 Dec 2023]. Available from: https://www.unitedagainstrabies.org/uar-zero-by-30-forum-12-01-2021-final/

[55] WOAH. Official disease status; 2022. Available from: https://www.woah.org/en/what-we-do/animal-health-and-welfare/official-disease-status/

[56] Lembo T, Hampson K, Haydon DT, Craft M, Dobson A, Dushoff J, et al. Exploring reservoir dynamics: a case study of rabies in the Serengeti ecosystem. J Appl Ecol. 2008;45(4):1246–57. doi: 10.1111/j.1365-2664.2008.01468.x 22427710 PMC3303133

[57] Vigilato MAN, Clavijo A, Knobl T, Silva HMT, Cosivi O, Schneider MC, et al. Progress towards eliminating canine rabies: policies and perspectives from Latin America and the Caribbean. Philos Trans R Soc Lond B Biol Sci. 2013;368(1623):20120143. doi: 10.1098/rstb.2012.0143 23798691 PMC3720041

[58] Fitzpatrick MC, Hampson K, Cleaveland S, Mzimbiri I, Lankester F, Lembo T, et al. Cost-effectiveness of canine vaccination to prevent human rabies in rural Tanzania. Ann Intern Med. 2014;160(2):91–100. doi: 10.7326/M13-0542 24592494 PMC4084874

[59] Putra AA, Hampson K, Girardi J, Hiby E, Knobel D, Mardiana IW, et al. Response to a rabies epidemic, Bali, Indonesia, 2008-2011. Emerg Infect Dis. 2013;19(4):648–51. 23632033 10.3201/eid1904.120380 PMC3647408

[60] Coleman PG, Dye C. Immunization coverage required to prevent outbreaks of dog rabies. Vaccine. 1996;14(3):185–6. doi: 10.1016/0264-410x(95)00197-9 8920697

[61] Tenzin, Wangdi K, Ward MP. Human and animal rabies prevention and control cost in Bhutan, 2001-2008: the cost-benefit of dog rabies elimination. Vaccine. 2012;31(1):260–70. doi: 10.1016/j.vaccine.2012.05.023 22634297

[62] Meltzer MI, Rupprecht CE. A review of the economics of the prevention and control of rabies. Part 2: Rabies in dogs, livestock and wildlife. Pharmacoeconomics. 1998;14(5):481–98. doi: 10.2165/00019053-199814050-00003 10344914

[63] Totton SC, Wandeler AI, Zinsstag J, Bauch CT, Ribble CS, Rosatte RC, et al. Stray dog population demographics in Jodhpur, India following a population control/rabies vaccination program. Prev Vet Med. 2010;97(1):51–7. doi: 10.1016/j.prevetmed.2010.07.009 20696487

[64] Mancy R, Rajeev M, Lugelo A, Brunker K, Cleaveland S, Ferguson EA, et al. Rabies shows how scale of transmission can enable acute infections to persist at low prevalence. Science. 2022;376(6592):512–6. doi: 10.1126/science.abn0713 35482879 PMC7613728

[65] Townsend SE, Sumantra IP, Pudjiatmoko, Bagus GN, Brum E, Cleaveland S, et al. Designing programs for eliminating canine rabies from islands: Bali, Indonesia as a case study. PLoS Negl Trop Dis. 2013;7(8):e2372. doi: 10.1371/journal.pntd.0002372 23991233 PMC3749988

[66] Cleaveland S, Lankester F, Townsend S, Lembo T, Hampson K. Rabies control and elimination: a test case for One Health. Vet Rec. 2014;175(8):188–93. doi: 10.1136/vr.g4996 25172649 PMC7612423

[67] Cleaveland S, Kaare M, Knobel D, Laurenson MK. Canine vaccination--providing broader benefits for disease control. Vet Microbiol. 2006;117(1):43–50. doi: 10.1016/j.vetmic.2006.04.009 16701966

[68] Zinsstag J, Schelling E, Roth F, Bonfoh B, de Savigny D, Tanner M. Human benefits of animal interventions for zoonosis control. Emerg Infect Dis. 2007;13(4):527–31. doi: 10.3201/eid1304.060381 17553265 PMC2725951

[69] Lv MM, Sun XD, Jin Z, Wu HR, Li MT, Sun GQ, et al. Dynamic analysis of rabies transmission and elimination in mainland China. One Health. 2023;17:100615. 37638210 10.1016/j.onehlt.2023.100615 PMC10458286

[70] Wallace RM, Cliquet F, Fehlner-Gardiner C, Fooks AR, Sabeta CT, Setién AA, et al. Role of oral rabies vaccines in the elimination of dog-mediated human rabies deaths. Emerg Infect Dis. 2020;26(12):1–9. doi: 10.3201/eid2612.201266 33219786 PMC7706920

[71] Cliquet F, Guiot A-L, Aubert M, Robardet E, Rupprecht CE, Meslin F-X. Oral vaccination of dogs: a well-studied and undervalued tool for achieving human and dog rabies elimination. Vet Res. 2018;49(1):61. doi: 10.1186/s13567-018-0554-6 30005701 PMC6045873

[72] Yale G, Lopes M, Isloor S, Head JR, Mazeri S, Gamble L. Review of oral rabies vaccination of dogs and its application in India. Viruses. 2022;14(1). 10.3390/v14010155 PMC8777998 35062358

[73] Fisher CR, Schnell MJ. New developments in rabies vaccination. Rev Sci Tech. 2018;37(2):657–72. doi: 10.20506/rst.37.2.2831 30747119

[74] Preiss S, Chanthavanich P, Chen LH, Marano C, Buchy P, van Hoorn R, et al. Post-exposure prophylaxis (PEP) for rabies with purified chick embryo cell vaccine: a systematic literature review and meta-analysis. Expert Rev Vaccines. 2018;17(6):525–45. doi: 10.1080/14760584.2018.1473765 29939085

[75] Faber M, Li J, Kean RB, Hooper DC, Alugupalli KR, Dietzschold B. Effective preexposure and postexposure prophylaxis of rabies with a highly attenuated recombinant rabies virus. Proc Natl Acad Sci U S A. 2009;106(27):11300–5. doi: 10.1073/pnas.0905640106 19581599 PMC2706273

[76] Li J, Liu Q, Liu J, Wu X, Lei Y, Li S, et al. An mRNA-based rabies vaccine induces strong protective immune responses in mice and dogs. Virol J. 2022;19(1):184. doi: 10.1186/s12985-022-01919-7 36371169 PMC9652961

[77] Gautret P, Shaw M, Gazin P, Soula G, Delmont J, Parola P, et al. Rabies postexposure prophylaxis in returned injured travelers from France, Australia, and New Zealand: a retrospective study. J Travel Med. 2008;15(1):25–30. doi: 10.1111/j.1708-8305.2007.00164.x 18217866

[78] Rupprecht CE, Briggs D, Brown CM, Franka R, Katz SL, Kerr HD, et al. Use of a reduced (4-dose) vaccine schedule for postexposure prophylaxis to prevent human rabies: recommendations of the advisory committee on immunization practices. MMWR Recomm Rep. 2010;59(RR-2):1–9. 20300058

[79] Overduin LA, Koopman JPR, Prins C, Verbeek-Menken PH, De Pijper CA, Eble PL. Boostability after single-visit pre-exposure prophylaxis with rabies vaccine: a randomised controlled non-inferiority trial. Lancet Infect Dis. 2023. 10.1016/S1473-3099(23)00452-8 37802090

[80] Guo C, Li Y, Huai Y, Rao CY, Lai S, Mu D. Exposure history, post-exposure prophylaxis use, and clinical characteristics of human rabies cases in China, 2006-2012. Sci Rep. 2018;8(1):17188. 30464190 10.1038/s41598-018-35158-0 PMC6249250

[81] Li D, Liu Q, Chen F, Jiang Q, Wang T, Yin X, et al. Knowledge, attitudes and practices regarding to rabies and its prevention and control among bite victims by suspected rabid animals in China. One Health. 2021;13:100264. doi: 10.1016/j.onehlt.2021.100264 34036144 PMC8135036

[82] Cliquet F, Aubert M, Sagné L. Development of a fluorescent antibody virus neutralisation test (FAVN test) for the quantitation of rabies-neutralising antibody. J Immunol Methods. 1998;212(1):79–87. doi: 10.1016/s0022-1759(97)00212-3 9671155

[83] Madhusudana SN, Malavalli BV, Thankappan UP, Sundramoorthy S, Belludi AY, Pulagumbaly SB, et al. Development and evaluation of a new immunohistochemistry-based test for the detection of rabies virus neutralizing antibodies. Hum Vaccin Immunother. 2014;10(5):1359–65. doi: 10.4161/hv.28042 24583787 PMC4896518

[84] Wang L, Zhang J, Meng S, Ge L, You Y, Xu Q, et al. Safety and immunogenicity of human rabies vaccine for the Chinese population after PEP: a systematic review and meta-analysis. Vaccine. 2022;40(32):4371–9. doi: 10.1016/j.vaccine.2022.06.035 35750539

[85] Minke JM, Bouvet J, Cliquet F, Wasniewski M, Guiot AL, Lemaitre L, et al. Comparison of antibody responses after vaccination with two inactivated rabies vaccines. Vet Microbiol. 2009;133(3):283–6. doi: 10.1016/j.vetmic.2008.06.024 18757142

[86] Klevar S, Høgåsen HR, Davidson RK, Hamnes IS, Treiberg Berndtsson L, Lund A. Cross-border transport of rescue dogs may spread rabies in Europe. Vet Rec. 2015;176(26):672. doi: 10.1136/vr.102909 26113337 PMC4501168

[87] Mansfield KL, Burr PD, Snodgrass DR, Sayers R, Fooks AR. Factors affecting the serological response of dogs and cats to rabies vaccination. Vet Rec. 2004;154(14):423–6. doi: 10.1136/vr.154.14.423 15119893

[88] Wei Y, Liu X, Li D, Chen S, Xu J, Chen K, et al. Canine rabies control and human exposure 1951-2015, Guangzhou, China. Bull World Health Organ. 2019;97(1):51–8. doi: 10.2471/BLT.18.217372 30618465 PMC6307510

[89] Wei Y, Li D, Yang Z, Chen K, Pan X, Xu J, et al. One Health responses to prevent the occurrence of rabies due to attacks by a rabid stray dog. Vet Med Sci. 2023;9(2):618–24. doi: 10.1002/vms3.986 36315409 PMC10029876

[90] Singh R, Singh KP, Cherian S, Saminathan M, Kapoor S, Manjunatha Reddy GB, et al. Rabies - epidemiology, pathogenesis, public health concerns and advances in diagnosis and control: a comprehensive review. Vet Q. 2017;37(1):212–51. 28643547 10.1080/01652176.2017.1343516

[91] Talbi C, Lemey P, Suchard MA, Abdelatif E, Elharrak M, Nourlil J. Phylodynamics and human-mediated dispersal of a zoonotic virus. PLoS Pathog. 2010;6(10):e1001166. 10.1371/journal.ppat.1001166 PMC2965766 21060816

[92] Lankau EW, Tack DM, Marano N. Crossing borders: one world, global health. Preventing rabies in an age of global travel. Clin Infect Dis. 2012;54(11):v–vi. doi: 10.1093/cid/cis429 22573903

[93] Tang X, Luo M, Zhang S, Fooks AR, Hu R, Tu C. Pivotal role of dogs in rabies transmission, China. Emerg Infect Dis. 2005;11(12):1970–2. 16485494 10.3201/eid1112.050271 PMC3367627

[94] Zhang Y, Vrancken B, Feng Y, Dellicour S, Yang Q, Yang W, et al. Cross-border spread, lineage displacement and evolutionary rate estimation of rabies virus in Yunnan Province, China. Virol J. 2017;14(1):102. doi: 10.1186/s12985-017-0769-6 28578663 PMC5457581

[95] Singh AJ, Chipman RB, de Fijter S, Gary R, Haskell MG, Kirby J, et al. Translocation of a stray cat infected with rabies from North Carolina to a terrestrial rabies-free county in Ohio, 2017. MMWR Morb Mortal Wkly Rep. 2018;67(42):1174–7. doi: 10.15585/mmwr.mm6742a2 30359345 PMC6290815

[96] Guo Z, Tao X, Yin C, Han N, Yu J, Li H, et al. National borders effectively halt the spread of rabies: the current rabies epidemic in China is dislocated from cases in neighboring countries. PLoS Negl Trop Dis. 2013;7(1):e2039. doi: 10.1371/journal.pntd.0002039 23383359 PMC3561166

[97] Trewby H, Nadin-Davis SA, Real LA, Biek R. Processes underlying rabies virus incursions across US-Canada border as revealed by whole-genome phylogeography. Emerg Infect Dis. 2017;23(9):1454–61. 28820138 10.3201/eid2309.170325 PMC5572885

[98] GOV.UK. Rabies risks in terrestrial animals by country; 202.3 Available from: https://www.gov.uk/government/publications/rabies-risks-by-country/rabies-risks-in-terrestrial-animals-by-country#k

[99] Taylor LH, Knopf L, Partners for rabies prevention. Surveillance of human rabies by national authorities—a global survey. Zoonoses Public Health. 2015;62(7):543–52. doi: 10.1111/zph.12183 25683444

[100] Scott TP, Coetzer A, Fahrion AS, Nel LH. Addressing the disconnect between the estimated, reported, and true rabies data: the development of a regional African Rabies Bulletin. Front Vet Sci. 2017;4:18. doi: 10.3389/fvets.2017.00018 28265562 PMC5316526

[101] Borse RH, Atkins CY, Gambhir M, Undurraga EA, Blanton JD, Kahn EB, et al. Cost-effectiveness of dog rabies vaccination programs in East Africa. PLoS Negl Trop Dis. 2018;12(5):e0006490. doi: 10.1371/journal.pntd.0006490 29791440 PMC5988334

[102] Minghui R, Stone M, Semedo MH, Nel L. New global strategic plan to eliminate dog-mediated rabies by 2030. Lancet Glob Health. 2018;6(8):e828–9. doi: 10.1016/S2214-109X(18)30302-4 29929890

[103] Bourhy H, Troupin C, Faye O, Meslin F-X, Abela-Ridder B, Sall AA, et al. Customized online and onsite training for rabies-control officers. Bull World Health Organ. 2015;93(7):503–6. doi: 10.2471/BLT.14.149849 26170509 PMC4490815

[104] Pieracci EG, Scott TP, Coetzer A, Athman M, Mutembei A, Kidane AH, et al. The formation of the eastern Africa rabies network: a sub-regional approach to rabies elimination. Trop Med Infect Dis. 2017;2(3):29. doi: 10.3390/tropicalmed2030029 28845466 PMC5568643

[105] Wu X, Hu R, Zhang Y, Dong G, Rupprecht CE. Reemerging rabies and lack of systemic surveillance in People’s Republic of China. Emerg Infect Dis. 2009;15(8):1159–64. doi: 10.3201/eid1508.081426 19751575 PMC2815959

[106] Rudd RJ, Smith JS, Yager PA, Orciari LA, Trimarchi CV. A need for standardized rabies-virus diagnostic procedures: effect of cover-glass mountant on the reliability of antigen detection by the fluorescent antibody test. Virus Res. 2005;111(1):83–8. doi: 10.1016/j.virusres.2005.03.014 15896406

[107] Mani RS, Madhusudana SN, Mahadevan A, Reddy V, Belludi AY, Shankar SK. Utility of real-time Taqman PCR for antemortem and postmortem diagnosis of human rabies. J Med Virol. 2014;86(10):1804–12. doi: 10.1002/jmv.23814 24136727

[108] Lodha L, Ananda AM, Ramachandran A, Manuel SP, Sannaiah SV, Mahadevan A, et al. Evaluation of a rapid, chip-based, micro-PCR assay for detection of rabies virus in human and canine specimens. J Med Virol. 2023;95(9):e29110. doi: 10.1002/jmv.29110 37728394

[109] Woodall J. Official versus unofficial outbreak reporting through the Internet. Int J Med Inform. 1997;47(1–2):31–4. doi: 10.1016/s1386-5056(97)00079-8 9506388

[110] Hartley DM, Nelson NP, Arthur RR, Barboza P, Collier N, Lightfoot N, et al. An overview of internet biosurveillance. Clin Microbiol Infect. 2013;19(11):1006–13. doi: 10.1111/1469-0691.12273 23789639

[111] Mtema Z, Changalucha J, Cleaveland S, Elias M, Ferguson HM, Halliday JEB, et al. Mobile phones as surveillance tools: implementing and evaluating a large-scale intersectoral surveillance system for rabies in Tanzania. PLoS Med. 2016;13(4):e1002002. doi: 10.1371/journal.pmed.1002002 27070315 PMC4829224

[112] Zhu Y, Lin S, Dong S, Zhang C, Shi L, Ren X. Incidence and trends of 17 notifiable bacterial infectious diseases in China, 2004-2019. BMC Infect Dis. 2023;23(1):273. 37131164 10.1186/s12879-023-08194-z PMC10152418

[113] Zhang Y-Z, Xiong C-L, Xiao D-L, Jiang R-J, Wang Z-X, Zhang L-Z, et al. Human rabies in China. Emerg Infect Dis. 2005;11(12):1983–4. doi: 10.3201/eid1112.040775 16485502 PMC3367615

[114] Song M, Tang Q, Rayner S, Tao X-Y, Li H, Guo Z-Y, et al. Human rabies surveillance and control in China, 2005-2012. BMC Infect Dis. 2014;14:212. doi: 10.1186/1471-2334-14-212 24742224 PMC4004447

[115] Miao F, Li N, Yang J, Chen T, Liu Y, Zhang S, et al. Neglected challenges in the control of animal rabies in China. One Health. 2021;12:100212. doi: 10.1016/j.onehlt.2021.100212 33553562 PMC7843516

[116] Zhou H, Vong S, Liu K, Li Y, Mu D, Wang L, et al. Human rabies in China, 1960-2014: a descriptive epidemiological study. PLoS Negl Trop Dis. 2016;10(8):e0004874. doi: 10.1371/journal.pntd.0004874 PMC4976867 27500957

[117] WHO. Zoonoses control—rabies situation and trends in Asia; 1997. [cited 2023]. Available from: https://www.who.int/publications/i/item/who-wer7235 9287498

[118] Chen Q, Liu Q, Gong C, Yin W, Mu D, Li Y, et al. Strategies to inTerrupt RAbies Transmission for the Elimination Goal by 2030 In China (STRATEGIC): a modelling study. BMC Med. 2023;21(1):100. doi: 10.1186/s12916-023-02821-x 36927437 PMC10022085

[119] Yin W, Fu ZF, Gao GF. Progress and prospects of dog-mediated rabies elimination in China. China CDC Wkly. 2021;3(39):831–4. doi: 10.46234/ccdcw2021.205 34595002 PMC8477050

[120] Qi L, Su K, Shen T, Tang W, Xiao B, Long J, et al. Epidemiological characteristics and post-exposure prophylaxis of human rabies in Chongqing, China, 2007-2016. BMC Infect Dis. 2018;18(1):6. doi: 10.1186/s12879-017-2830-x 29295708 PMC5751830

[121] Hu RL, Fooks AR, Zhang SF, Liu Y, Zhang F. Inferior rabies vaccine quality and low immunization coverage in dogs (Canis familiaris) in China. Epidemiol Infect. 2008;136(11):1556–63. doi: 10.1017/S0950268807000131 18177524 PMC2870756

[122] Si H, Guo Z-M, Hao Y-T, Liu Y-G, Zhang D-M, Rao S-Q, et al. Rabies trend in China (1990-2007) and post-exposure prophylaxis in the Guangdong province. BMC Infect Dis. 2008;8:113. doi: 10.1186/1471-2334-8-113 18717989 PMC2532688

[123] Yin C, Zhou H, Wu H, Tao X, Rayner S, Wang S, et al. Analysis on factors related to rabies epidemic in China from 2007-2011. Virol Sin. 2012;27(2):132–43. doi: 10.1007/s12250-012-3244-y 22492004 PMC8218126

[124] Tan J, Wang R, Ji S, Nanjing Agricultural University research group of The Challenge Cup Rabies Research Group, Su S, Zhou J. One Health strategies for rabies control in rural areas of China. Lancet Infect Dis. 2017;17(4):365–7. doi: 10.1016/S1473-3099(17)30116-0 28262599

[125] Fisman DN. Taking the bite out of rabies, putting teeth into “One Health”. Ann Intern Med. 2014;160(2):132–3. doi: 10.7326/M13-2821 24592498

[126] Perez de Diego AC, Vigo M, Monsalve J, Escudero A. The One Health approach for the management of an imported case of rabies in mainland Spain in 2013. Euro Surveill. 2015;20(6). 10.2807/1560-7917.es2015.20.6.21033 25695478

[127] WHO. WHO expert consultation on rabies: third report; 2018. Available from: http://apps.who.int/iris/handle/10665/272364

[128] Chomel BB, Trotignon J. Epidemiologic surveys of dog and cat bites in the Lyon area, France. Eur J Epidemiol. 1992;8(4):619–24. doi: 10.1007/BF00146385 1397233

[129] Crowcroft NS, Thampi N. The prevention and management of rabies. BMJ. 2015;350:g7827. doi: 10.1136/bmj.g7827 25589091

